# Supplementary material for: Altered microRNA Transcriptome in Cultured Human Liver Cells upon Infection with Ebola Virus
Source: Int J Mol Sci. 2021 Apr 6;22(7):3792. doi: 10.3390/ijms22073792 (PMC8038836; doi:10.3390/ijms22073792)
Supplement: Supplementary file 1 [file ijms-22-03792-s001.zip › Supplementary_File/C_ GO_Analysis_Results/16-30nt_go_Makona-96h-Huh7_vs_Control-96h-Huh7_up.mature_mirna_targets/BP_FoldEnrichmentDotPlot.pdf]

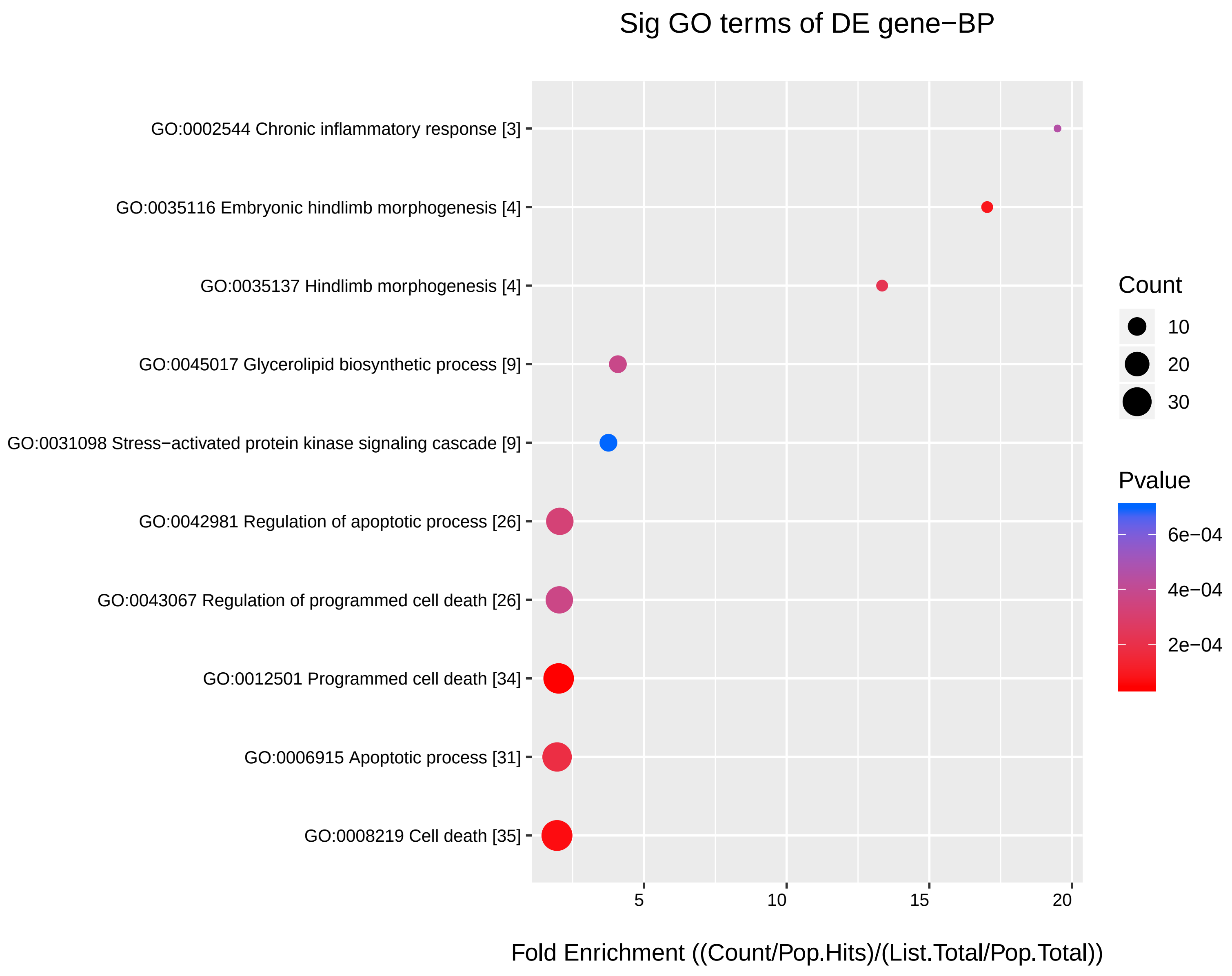
Sig GO terms of DE gene−BP
GO:0002544 Chronic inflammatory response [3]
GO:0035116 Embryonic hindlimb morphogenesis [4]
GO:0035137 Hindlimb morphogenesis [4]
GO:0045017 Glycerolipid biosynthetic process [9]
GO:0031098 Stress−activated protein kinase signaling cascade [9]
GO:0042981 Regulation of apoptotic process [26]
GO:0043067 Regulation of programmed cell death [26]
GO:0012501 Programmed cell death [34]
GO:0006915 Apoptotic process [31]
GO:0008219 Cell death [35]
5
10
15
20
Fold Enrichment ((Count/Pop.Hits)/(List.Total/Pop.Total))
Count
10
20
30
Pvalue
6e−04
4e−04
2e−04
